# Zinc-responsive coactivator recruitment by the yeast Zap1 transcription factor

**DOI:** 10.1002/mbo3.8

**Published:** 2012-06

**Authors:** Avery G Frey, David J Eide

**Affiliations:** Department of Nutritional Sciences, University of Wisconsin-MadisonMadison, Wisconsin

**Keywords:** Coactivators, homeostasis, regulation, yeast, Zap1 transcription, zinc

## Abstract

The zinc-responsive Zap1 transcription factor of *Saccharomyces cerevisiae* controls many genes involved in zinc homeostasis. Zap1 has two activation domains, AD1 and AD2, which are independently regulated by zinc. While AD1 can fully activate most Zap1 target genes, AD2 is active only on a subset of those genes. One hypothesis explaining this promoter specificity was that AD1 and AD2 recruit different coactivators. To address this question, we carried out a genetic screen to identify coactivator complexes that are required for Zap1-mediated activation. SWI/SNF, SAGA, and Mediator complexes were implicated as playing major roles in Zap1 activation. Consistent with this conclusion, we found that these three complexes are recruited to Zap1 target promoters in a zinc-responsive and Zap1-dependent manner. Coactivator recruitment was highly interdependent such that mutations disrupting SAGA impaired recruitment of SWI/SNF and vice versa. Optimal Mediator recruitment was dependent on both SAGA and SWI/SNF. A comparison of the coactivators recruited by AD1 and AD2 found no obvious differences suggesting that recruitment of different coactivators is not likely the mechanism of AD specificity. Rather, our results suggest that AD2 recruits coactivators less effectively than AD1 and is therefore only functional on some promoters.

## Introduction

Eukaryotic transcriptional activators function by recruiting proteins and protein complexes, termed “coactivators”, to target promoters. These coactivators facilitate transcription initiation through a variety of mechanisms. Some coactivators, such as the SWI/SNF, ISW1, and RSC complexes, have ATP-dependent chromatin remodeling activity and aid transcriptional activation by clearing promoter regions of inhibitory nucleosomes (Martens and Winston, 2003). Other coactivators, such as the SAGA, ADA, and NuA4 complexes, have acetyl transferase (HAT) activity and modify histones in nucleosomes to diminish their DNA packaging capacity (Sterner and Berger, 2000). Other coactivator complexes serve as bridging adaptors between activator proteins and RNA polymerase II. The Mediator and TFIID complexes are well-known examples of this type of coactivator (Biddick and Young, 2005; Bhaumik, 2011). Transcriptional activation often requires a combination of these factors to be recruited to a promoter (Biddick and Young, 2009).

In this report, we characterize the coactivator requirements of the zinc-responsive Zap1 transcription factor of *Saccharomyces cerevisiae*. Zap1 is the central player in yeast zinc homeostasis because it activates expression of as many as 80 genes in zinc-limited cells (Eide, 2009). Genes induced by Zap1 encode proteins such as the plasma membrane zinc transporters Zrt1, Zrt2, and Fet4, the vacuolar zinc transporters Zrt3 and Zrc1, and other proteins important to adapting to zinc deficiency conditions (Lyons et al., 2000; Wu et al., 2008). Zap1 binds to one or more 11 bp zinc-responsive elements (ZRE) in the promoters of its target genes. The consensus sequence for the ZRE is 5′-ACCTTNAAGGT-3′.

**Figure 1:**
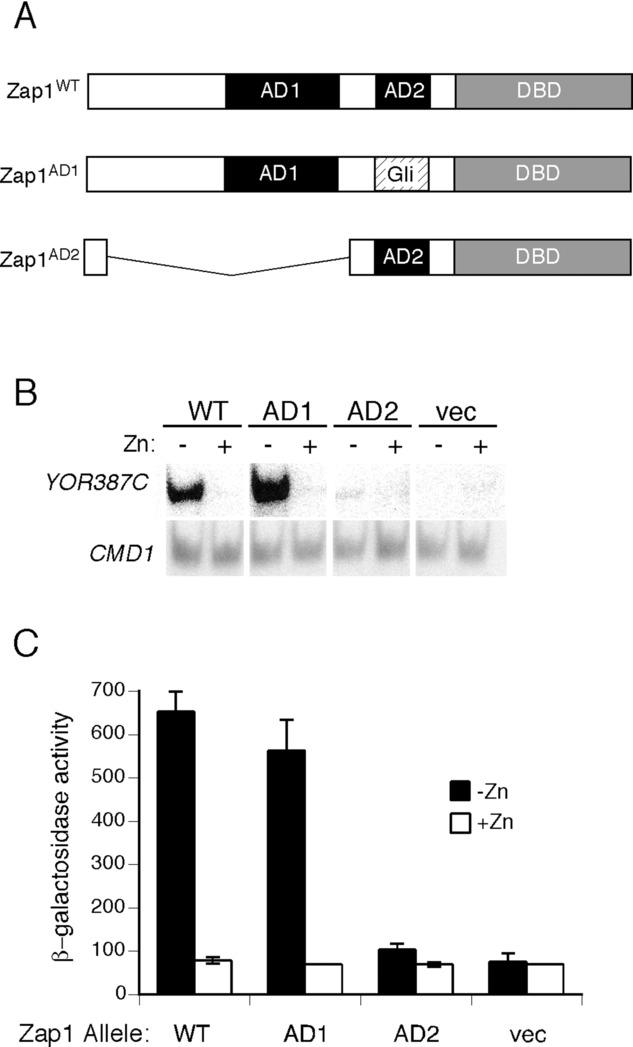
*YOR387C* induction by Zap1 is AD1 specific. (A) Zap1 alleles used in this study. (B) *zap1Δ* cells expressing either Zap1^WT^, Zap1^AD1^, Zap1^AD2^, or the empty vector were grown to mid-log phase in LZM supplemented with either 1 μM (−) or 1000 μM (+) ZnCl_2_. Total RNA was then isolated and *YOR387C* mRNA levels were measured by S1 nuclease protection assays. Expression of *CMD1*, which encodes calmodulin, was used as a loading control. (C) Cells as described in panel B bearing the *YOR387C-lacZ* reporter were grown to mid-log phase in LZM supplemented with either 1 μM (*filled* columns) or 1000 μM (*open* columns) ZnCl_2_. Cells were then harvested, and β-galactosidase assays were performed. The values shown are the means of three independent cultures and the error bars represent ±1 SD.

Zinc regulates Zap1 activity through at least four mechanisms. First, Zap1 regulates its own transcription by positive transcriptional autoregulation (Zhao and Eide, 1997). Zinc-limited cells increase the level of Zap1 and this increase may facilitate activation of target genes with weaker ZREs (Wu et al., 2008). The Zap1 DNA-binding domain contains five C_2_H_2_ zinc fingers (Znf3-7), each of which is required for DNA-binding activity (Fig. 1A) (Evans-Galea et al., 2003). The interaction of this domain with ZREs in vivo is regulated by zinc status such that binding is reduced in zinc-replete cells (Frey et al., 2011). Although the mechanism of this regulation is not yet known, it does not appear to involve the direct binding of zinc to the DNA binding domain to disrupt Zap1 binding.

Zap1 also contains two activation domains whose functions are regulated by zinc. Activation domain 1 (AD1) was mapped to residues 207–402 (Fig. 1A) (Frey and Eide, 2011). This region is found within a larger zinc-responsive domain, ZRD^AD1^, which spans residues 182–502 (Herbig et al., 2005). Current evidence suggests that zinc binding to ZRD^AD1^ causes a conformational change in the protein that masks the ability of AD1 to activate transcription. A similar mechanism controls activation domain 2 (AD2). AD2 maps to residues 611–640 and is found within a second zinc-responsive domain (ZRD^AD2^) defined by two zinc fingers, Znf1 and Znf2 (Bird et al., 2003; Wang et al., 2006). Zinc binding to those zinc fingers represses AD2 function. We have previously proposed that zinc binding to ZRD^AD1^ and the zinc fingers of ZRD^AD2^ inhibits coactivator recruitment by AD1 and AD2 (Bird et al., 2003; Herbig et al., 2005).

A remarkable feature of Zap1's two activation domains is that they are regulated by zinc independently of each other. Moreover, both domains are evolutionarily conserved in Zap1 orthologs from widely divergent fungal species. These observations suggested that the two Zap1 activation domains play distinct roles in zinc-limited cells. We recently addressed those functions by examining Zap1 target gene expression in cells expressing wild-type Zap1 or Zap1 alleles with only AD1 or AD2 (Frey and Eide, 2011). Our results indicated that AD1 plays the primary role on all Zap1-regulated promoters. With respect to AD2 function, Zap1 target genes fall into two general classes, those genes that are efficiently activated by AD2 and those on which AD2 is inactive. To explain these results, one hypothesis we proposed was that AD1 and AD2 recruit different coactivators and thus their ability to activate a given promoter depends on the contribution that their respective coactivators can make to target gene induction. To test this model and better understand how Zap1 activates transcription in response to low zinc, we conducted a genetic screen to identify coactivators recruited by AD1 that are required for Zap1 activity. The results indicated that Zap1 recruits SWI/SNF, SAGA, and Mediator to its target promoters and that this recruitment is Zap1 dependent and zinc responsive. Both AD1 and AD2 showed similar coactivator requirements suggesting that these domains do not recruit different coactivators. Rather, our results indicate that AD2 is a weaker domain than AD1 and this may explain why it is less able to activate some promoters.

## Results

### A genetic screen to identify coactivator complexes required for Zap1 AD1 function

To characterize the coactivator requirements of Zap1, we focused initially on identifying those coactivator proteins and complexes specifically required for AD1 function. This approach was feasible because some genes are completely dependent on AD1 and AD2 does not contribute to their induction in low zinc. Our previous studies suggested that *YOR387C* is one such gene (Frey and Eide, 2011). The product of *YOR387C* is a cell wall protein of unknown function, but this gene is a highly induced Zap1 target.

The Zap1 alleles used to assess the AD specificity of *YOR387C* activation are depicted in Figure 1A. Each allele has six amino-terminal myc epitope tags and was expressed from a plasmid vector at a low constitutive level similar to that of chromosomally expressed Zap1 (Frey and Eide, 2011). The first allele, designated “Zap1^WT^”, contained full-length Zap1. In the second allele, referred to as “Zap1^AD1^”, the Znf1 and Znf2 zinc fingers of Zap1 (residues 581–641) were deleted and replaced with Znf1 and Znf2 from the human Gli protein. The Gli Znf1/Znf2 domain lacks any detectable activation domain function (Frey and Eide, 2011). Thus, this substitution removes all AD2 function by replacing that region with a structurally similar but transcriptionally inactive domain. The third allele was “Zap1^AD2^” in which amino acids 6–551 were deleted. Because the structure of AD1 is unknown, we knew of no structural ortholog of this domain and were unable to generate an allele analogous to the Gli substitution in Zap1^AD1^. Nonetheless, given the extent of the deletion, Zap1^AD2^ lacks all AD1 function.

A *zap1Δ* mutant expressing Zap1^AD1^ was capable of fully inducing chromosomal *YOR387C* mRNA expression in a low zinc culture medium while Zap1^AD2^ was unable to activate transcription of this gene (Fig. 1B). When the *YOR387C* promoter was fused to the *lacZ* reporter gene and assayed for expression using β-galactosidase activity assays, the plasmid-born *YOR387C-lacZ* reporter was regulated in the same manner as the chromosomal promoter (Fig. 1C).

Because the *YOR387C-lacZ* fusion showed the same specificity for AD1 function as the chromosomal gene, we used this reporter to test a large number of mutants disrupted for different coactivator subunits for their ability to drive its expression. Fifty-eight different coactivator mutant strains from the yeast deletion collection were transformed with the *YOR387C-lacZ* reporter, grown in low zinc, and then assayed for β-galactosidase activity (Table 1). Activities measured ranged from 6–181% of the activity observed in the isogenic wild-type strain. Several mutants showed decreased *YOR387C-lacZ* expression relative to the wild-type strain suggesting that the affected coactivators may be required for Zap1 function.

**Table 1:** Effects of coactivator mutations on *YOR387C-lacZ* expression

Strain	Coactivator complex	Percentage of WT expression^1^
Wild-type (BY4743)	**-**	100
***zap1Δ***	**-**	**14**
*ahc1Δ*	ADA	106
***ada2Δ***	**ADA, SAGA**	**17**
***ada3Δ***	**ADA, SAGA**	**36**
*ada1Δ*	SAGA	NA^2^
*ada5Δ*	SAGA	NA^2^
***spt3Δ***	**SAGA**	**14**
*spt7Δ*	SAGA	135
*spt8Δ*	SAGA	102
*chd1Δ*	SAGA	86
*yer049wΔ*	NuA3	95
*sas3Δ*	NuA3	113
*eaf3Δ*	NuA4	149
*bdf1Δ*	TFIID	53
*bdf2Δ*	TFIID	95
*elp3Δ*	HAT	89
*ayt1Δ*	HAT	66
*hpa2Δ*	HAT	95
*hpa3Δ*	HAT	100
*sas2Δ*	HAT	111
*taf14Δ*	TFIID, SWI/SNF, NuA3	102
*snf2Δ*	SWI/SNF	115
***swi3Δ***	**SWI/SNF**	**21**
*snf5Δ*	SWI/SNF	85
***snf6Δ***	**SWI/SNF**	**24**
*snf11Δ*	SWI/SNF	105
*rsc1Δ*	RSC	98
***rsc2Δ***	**RSC**	**41**
*isw1Δ*	ISW1	92
*isw2Δ*	ISW2	103
*itc1Δ*	ISW2	89
*not3Δ*	CCR4-NOT	181
*not4Δ*	CCR4-NOT	115
***not5Δ***	**CCR4-NOT**	**11**
*caf1Δ*	CCR4-NOT	172
*caf4Δ*	CCR4-NOT	135
*caf16Δ*	CCR4-NOT	115
*caf40Δ*	CCR4-NOT	62
*caf130Δ*	CCR4-NOT	113
***dhh1Δ***	**CCR4-NOT**	**6**
*ssn3Δ*	Mediator/SRB	70
*ssn8Δ*	Mediator/SRB	78
*srb2Δ*	Mediator/SRB	131
*srb8Δ*	Mediator/SRB	157
*med1Δ*	Mediator/SRB	73
*nut1Δ*	Mediator/SRB	111
*pgd1Δ*	Mediator/SRB	117
*sin4Δ*	Mediator/SRB	68
*med15Δ*	Mediator/SRB	NA^2^
*cdc73Δ*	PAF1	93
*rtf1Δ*	PAF1	87
*leo1Δ*	PAF1	65
*paf1Δ*	PAF1	NA^2^
***ccr4Δ***	**CCR4-NOT, PAF1**	**24**
*hpr1Δ*	THO/TREX	86
***mft1Δ***	**THO/TREX**	**16**
*tex1Δ*	THO/TREX	100
*thp2Δ*	THO/TREX	96
*dst1Δ*	TFIIS	119

1 Values below 50% of wild-type expression are shown in bold.

2 NA, not assayed due to poor growth in low zinc.

We chose an arbitrary cut-off value and focused on those mutants with more than a 50% decrease in expression relative to wild-type levels. We predicted that these subunits, and the complexes in which they are found, are most important for Zap1 AD1 function. As a result, we identified nine mutants with strong defects in *YOR387C-lacZ* expression. These mutations affected components of SAGA and related complexes (*ada2Δ*, *ada3Δ*, *spt3Δ*), SWI/SNF (*swi3Δ*, *snf6Δ*), RSC (*rsc2Δ*), CCR4-NOT (*ccr4Δ*, *dhh1Δ*, *not5Δ*), and THO-TREX (*mft1Δ*) complexes. In addition, we identified four other coactivator subunit mutants that grew very poorly in low zinc and therefore could not be assayed. Given that Zap1 activity is required for low zinc growth, these mutated genes may encode additional candidates for Zap1-recruited coactivators. The mutants that grew too poorly in low zinc to assay were *ada1Δ* and *ada5Δ* (SAGA complex), *med15Δ* (Mediator), and *paf1Δ* (PAF1 complex).

**Figure 2:**
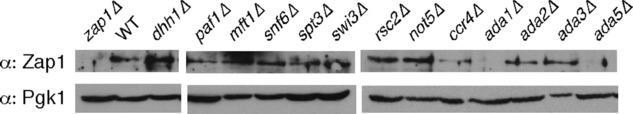
Zap1 protein accumulation is not affected in most coactivator mutant strains. Coactivator deletion mutants that affected *YOR387C-lacZ* expression were grown to mid-log phase in LZM supplemented with 1 μM ZnCl_2._ Protein extracts were prepared and analyzed by immunoblotting using an antibody raised against the DNA binding domain of Zap1. Pgk1 phosphoglycerate kinase was used as a loading control.

The results of this screen suggested that Zap1 directly or indirectly recruits ATP-dependent chromatin remodeling complexes (SWI/SNF, RSC), the SAGA histone acetylating complex, and possibly the Mediator complex to activate the *YOR387C* promoter. An alternative hypothesis was that the defect in expression of *YOR387C-lacZ* was due to impaired activation of the *ZAP1* promoter thereby depleting the cell of Zap1 and indirectly reducing *YOR387C* expression. To test this possibility, we assayed Zap1 levels in wild-type cells and coactivator mutants by immunoblotting. Levels of Zap1 protein similar to that of wild-type cells were observed in all coactivator mutants tested except *ccr4Δ*, *ada1Δ*, and *ada5Δ* (Fig. 2). The very low level of Zap1 in *ada1Δ* and *ada5Δ* strains provides a clear explanation for the inability of these cells to grow in zinc-limiting media where Zap1 activity is required. Notably, *ccr4Δ* mutants also grow poorly in low zinc (data not shown).

### Coactivators required for activation of YOR387C-*lacZ* are not activation domain specific

**Figure 3:**
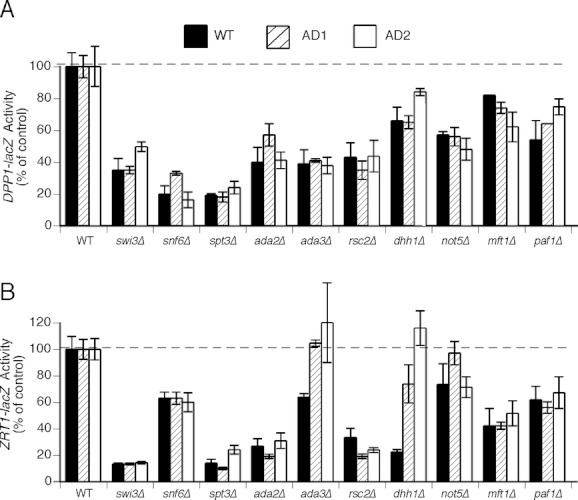
Coactivators required for activation of *YOR387C-lacZ* are not activation domain specific. The indicated coactivator mutant strains lacking chromosomal *ZAP1* (*zap1Δ*) and expressing either Zap1^WT^ (*filled* columns), Zap1^AD1^ (*hatched* columns), or Zap1^AD2^ (*open* columns) were transformed with *DPP1-lacZ* (A) or *ZRT1-lacZ* (B) reporters. These cells were grown to mid-log phase in LZM supplemented with 1 μM ZnCl_2_ and then assayed for β-galactosidase activity. The shown values are the means of three independent cultures and are expressed as a percentage of the isogenic *zap1Δ* strain expressing the corresponding Zap1 allele but lacking any coactivator mutation. The values shown are the means of three independent cultures and the error bars represent ±1 SD. The dashed line indicates 100% activity measured in the control strains.

To assess whether the coactivator subunits identified in the genetic screen were specifically required for AD1-activated transcription or might also mediate transcription activation by AD2, we assessed Zap1 activity in those coactivator mutants using two promoters that can be activated by either AD1 and AD2, that is *DPP1* and *ZRT1* (Frey and Eide, 2011). *ZRT1* encodes a zinc uptake transporter and *DPP1* encodes diacylglycerol pyrophosphate phosphatase that is induced in low zinc for unknown reasons. Coactivator mutant strains lacking chromosomal *ZAP1* (*zap1Δ*) and expressing either Zap1^WT^, Zap1^AD1^, or Zap^AD2^ were transformed with *DPP1-lacZ* or *ZRT1-lacZ* reporters. These cells were grown in zinc-limiting conditions and assayed for β-galactosidase activity. Expression of the *DPP1-lacZ* reporter was reduced in all coactivator mutants tested relative to the corresponding wild-type control strain (Fig. 3A). Moreover, the effect of each coactivator mutation was similar in cells expressing Zap1^WT^, Zap1^AD1^, and Zap1^AD2^ alleles. This result suggests that on this promoter, AD1 and AD2 are similarly reliant on these coactivators. Most highly affected were mutants affecting subunits of the SWI/SNF (*swi3Δ, snf6Δ*) and SAGA (*spt3Δ, ada2Δ, ada3Δ*) complexes. Results obtained with the *ZRT1-lacZ* reporter were similar (Fig. 3B) although Zap1^WT^ appeared to be more severely affected by loss of Ada3 and Dhh1 than either Zap1^AD1^ or Zap1^AD2^. The reason for this effect is unknown.

### Zinc- and Zap1-dependent recruitment of coactivator complexes

**Figure 4:**
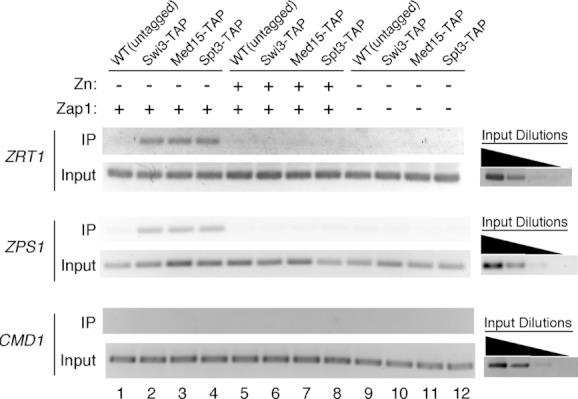
Recruitment of Swi3, Med15, and Spt3 is zinc responsive and Zap1 dependent. Untagged wild-type (BY4741), and isogenic Swi3-TAP, Med15-TAP, and Spt3-TAP tagged strains were grown in LZM supplemented with either 1 μM (−, lanes 1–4) or 1000 μM (+, lanes 5–8) ZnCl_2_. Isogenic *zap1Δ* cells grown in low zinc were used in lanes 9–12. The cells were cross-linked with formaldehyde, harvested, and chromatin immunoprecipitation analysis was performed using IgG-Sepharose to immunoprecipitate the TAP-tagged proteins. Coprecipitation of specific DNA fragments was then assessed by PCR using primers flanking the ZREs of the *ZRT1* and *ZPS1* promoters. Primers specific for the *CMD1* promoter were used as a negative control. PCR amplification of 10-fold serial dilutions of input samples was used to confirm the quantitative nature of the assay.

The results of Figure 3 suggested that AD1 and AD2 are both highly reliant on SWI/SNF and SAGA and may recruit those complexes to Zap1 target promoters in a Zap1-dependent and zinc-responsive manner. To test this hypothesis directly, we assayed recruitment of these complexes to the *ZRT1* promoter in vivo using chromatin immunoprecipitation. Recruitment of Mediator complex was also tested because the *med15Δ* mutant was unable to grow in low zinc, thereby suggesting a role for Mediator in the Zap1 activation process. PCR analysis of input DNAs indicated similar levels of total chromatin were used in each immunoprecipitation and the quantitativeness of the assay was confirmed using serial dilutions of input DNA samples (Fig. 4). Immunoprecipitation of TAP-tagged Swi3 (SWI/SNF), Spt3 (SAGA), and Med15 (Mediator) from cross-linked chromatin isolated from zinc-limited cells all resulted in enrichment of the *ZRT1* promoter (Fig. 4, lanes 1–4). These results indicate recruitment of these subunits and their complexes occurs in zinc-limited wild-type cells. This recruitment was zinc responsive as it was not observed in zinc-replete cells (Fig. 4, lanes 5–8). Moreover, this recruitment was Zap1 dependent because it was not observed in *zap1Δ* mutant cells grown in low zinc (Fig. 4, lanes 9–12). Similar results were obtained with the *ZPS1* promoter; *ZPS1* encodes a cell wall protein of unknown function and is also a Zap1 target gene. Chromatin immunoprecipitation of the *CMD1* promoter was used as a negative control to confirm that the effects observed with the *ZRT1* and *ZPS1* promoters were specific. *CMD1* is known to be dependent on TFIID recruitment and not dependent on the coactivators examined in this assay (Huisinga and Pugh, 2004). As expected, no enrichment of *CMD1* promoter DNA was observed.

### Interdependence of coactivator recruitment by Zap1

**Figure 5:**
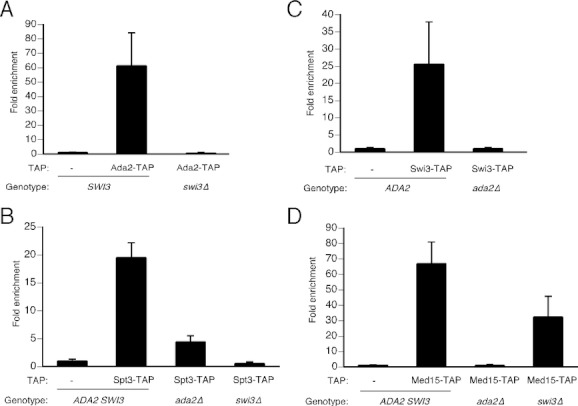
Coactivator recruitment by Zap1 is strongly interdependent. Results from chromatin immunoprecipitation of Ada2-TAP (A), Spt3-TAP (B), Swi3-TAP (C), and Med15-TAP (D) are shown. Isogenic *ada2Δ* and *swi3Δ* mutants were used to determine the effects of disrupting one complex on the recruitment of others. The indicated strains were grown to mid-log phase in LZM supplemented with 1 μM ZnCl_2_. Cells were then cross-linked with formaldehyde, harvested, and chromatin immunoprecipitation analysis was performed using IgG-Sepharose to immunoprecipitate TAP-tagged proteins. Coprecipitation of specific DNA fragments was then assessed by real-time PCR using primers flanking the ZREs of the *ZRT1* promoter. The values shown are the means of three independent cultures and the error bars represent 1 SD.

Coactivator recruitment at promoters can be ordered such that disrupted recruitment at early steps can alter recruitment of other coactivators later in the assembly process (Biddick and Young, 2009). Alternatively, larger multi-coactivator super-complexes may be destabilized by the absence of one coactivator component regardless of the order of recruitment. To determine the possible interdependence of coactivator recruitment by Zap1, we examined whether disruption of one complex affected recruitment of other complexes. Chromatin immunoprecipitation of the *ZRT1* promoter was used to assess the interdependency of coactivator recruitment. First, we found that lack of SWI/SNF complex subunit Swi3 disrupted recruitment of SAGA to *ZRT1* when assayed using Ada2-TAP (Fig. 5A). This result was confirmed using chromatin immunoprecipitation of a second SAGA subunit, Spt3. Immunoprecipitation of the *ZRT1* promoter with Spt3-TAP was reduced in the *ada2Δ* mutant, consistent with both Spt3 and Ada2 being components of SAGA (Fig. 5B). Spt3-TAP immunoprecipitation of the *ZRT1* promoter was eliminated in the *swi3Δ* mutant indicating again that SAGA recruitment requires SWI/SNF function. The converse was also true. Swi3-TAP immunoprecipitation of the *ZRT1* promoter was completely inhibited in the *ada2Δ* SAGA complex mutant (Fig. 5C). Similarly, recruitment of Mediator was impaired in both the *swi3Δ* and the *ada2Δ* mutants (Fig. 5D). These results suggest that efficient Mediator recruitment to *ZRT1* is dependent on both SWI/SNF and SAGA. Thus, SWI/SNF, SAGA, and Mediator recruitment to the *ZRT1* promoter appears to be highly interdependent.

### Coactivator recruitment efficiency suggests that AD2 is weaker than AD1

**Figure 6:**
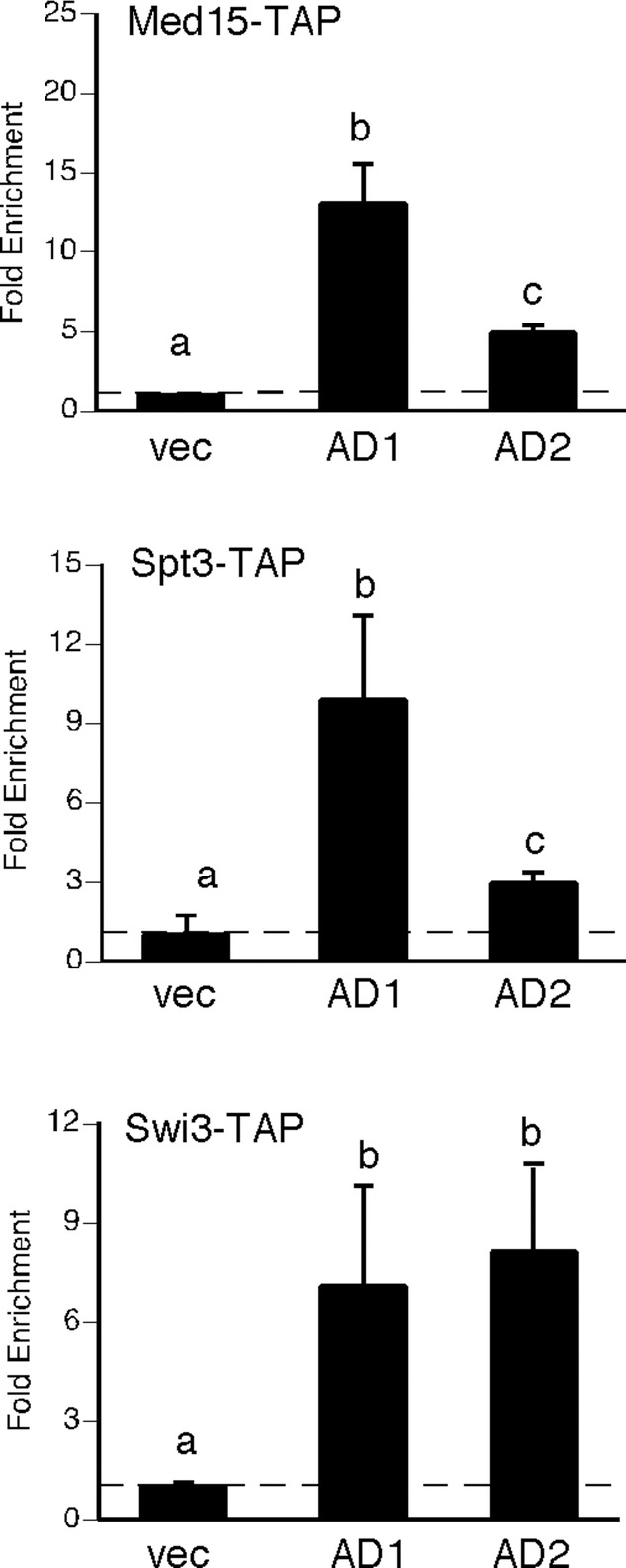
Coactivator recruitment by AD2 is weaker than AD1-mediated recruitment. Chromosomal *ZAP1* was deleted from strains expressing Med15-TAP, Spt3-TAP, or Swi3-TAP. The resulting strains were transformed with Zap1^AD1^, Zap1^AD2^, or the empty vector and grown to mid-log phase in LZM supplemented with 1 μM ZnCl_2_. These cells were then cross-linked with formaldehyde, harvested, and chromatin immunoprecipitation analysis was performed using IgG-Sepharose to immunoprecipitate TAP-tagged proteins. Coprecipitation of the *ZRT1* promoter was then assessed by real-time PCR using primers flanking the *ZRT1* ZREs. The values shown are the means of three independent cultures and the error bars represent 1 SD. The letters denote values that are significantly different from each other (*P* < 0.05) as determined using ANOVA. The dashed lines mark the background level of recruitment observed in the vector-only cells.

The genetic results shown in Figure 3 suggest that AD1 and AD2 both recruit similar coactivators to Zap1-responsive promoters. To test this hypothesis directly, we examined coactivator recruitment by AD1 and AD2 alone using chromatin immunoprecipitation. On the chromosomal *ZRT1* promoter, a promoter that is efficiently activated by either AD1 or AD2, we found that immunoprecipitation of Med15-TAP, Spt3-TAP, and Swi3-TAP resulted in enrichment of that promoter DNA from cells expressing Zap1 alleles bearing AD1 or AD2 alone (Fig. 6). Recruitment of Med15-TAP and Spt3-TAP by Zap1^AD2^ was lower than their recruitment by Zap1^AD1^ suggesting that AD2 is less able to recruit those factors than AD1. Both AD1 and AD2 were equally capable of Swi3-TAP recruitment. These results support the hypothesis that both AD1 and AD2 recruit SWI/SNF, SAGA, and Mediator, but suggest that AD2 is less effective in certain aspects of this recruitment than AD1.

## Discussion

In this study, we initiated a characterization of the coactivator requirements of the Zap1 transcription factor. A genetic screen was used to identify coactivator complex subunits required for optimal expression of one Zap1 target gene, *YOR387C*, in low zinc. A similar approach was used previously to identify coactivators used by the Gcn4 transcription factor (Swanson et al., 2003). Among the approximately 60 coactivator mutants that we analyzed, 10 were found to have reduced *YOR387C* expression below an arbitrary threshold value of 50%. The identity of those mutants implicated SWI/SNF and SAGA complexes as being particularly important for Zap1-induced expression. In addition, given the very poor growth of the *med15Δ* mutant in low zinc, Mediator complex was also implicated. Other complexes that we identified that may play lesser roles on Zap1-regulated genes include CCR4-NOT, THO-TREX, and Paf1 complexes. These complexes play various roles in transcription initiation, transcription elongation, RNA export, and RNA degradation (Collart, 2003; Jaehning, 2010; Rondon et al., 2010).

We focused our attention on the SWI/SNF, SAGA, and Mediator complexes because of the strong effect mutations affecting those complexes had on *YOR387C* expression. It should be noted, however, that our genetic screen was not exhaustive and many more coactivator mutants await analysis. In addition, other coactivator mutants that we found to reduce *YOR387C* expression but not below our threshold value may also affect coactivator complexes that are recruited by Zap1 and contribute significantly to transcription. Thus, while we have identified coactivator complexes important for Zap1 function, other unrecognized coactivators may be involved as well.

Alternative approaches to identifying coactivators recruited by a given transcription factor include chromatin immunoprecipitation coupled with mass spectrometry to identify interacting proteins. While this approach has been used successfully by others, we believe that the genetic approach we have used has a significant advantage. Our approach can identify coactivator complexes that are not only recruited to a promoter but must also play significant functional roles in the initiation process. While the genetic approach does not specifically identify coactivators that interact directly with Zap1, it does highlight those factors that are especially important for gene expression whether they are directly or indirectly recruited by the Zap1 activation domains.

The initial goal of our genetic screen was to identify those factors required for AD1 function. This was possible because *YOR387C* transcription is entirely dependent on AD1 and AD2 does not activate this promoter. Thus, mutations found to decrease *YOR387C-lacZ* expression are likely to affect AD1-mediated activation. Once identified, we could then test the role of these factors on other Zap1-regulated promoters. By analyzing the *DPP1* and *ZRT1* promoters, we found that the effects of the coactivator mutants were similar. These results suggest that at least for the *YOR387C*, *DPP1*, and *ZRT1* promoters, Zap1-mediated activation has similar coactivator requirements. Because *DPP1* and *ZRT1* can be activated by either AD1 or AD2, we could also compare the effects of these mutations on either AD1 or AD2 function. We found that the effects of these mutations on these two activation domains were also similar. These results suggest that AD1 and AD2 have similar coactivator requirements. This hypothesis was supported by chromatin immunoprecipitation experiments showing that AD1 and AD2 can both recruit SWI/SNF, SAGA, and Mediator to a Zap1 target promoter.

We began this study with the hypothesis that, in zinc-replete cells, zinc binding to ligand residues within and flanking AD1 and AD2 blocks coactivator recruitment (Bird et al., 2003; Herbig et al., 2005). This was confirmed when we examined the zinc and Zap1 dependence of coactivator recruitment. Assembly of coactivators on Zap1 target promoters was also found to be highly interdependent with SAGA recruitment requiring SWI/SNF activity and vice versa. Optimal recruitment of Mediator complex was also dependent on both SWI/SNF and SAGA function. From our results, we cannot assess the order of recruitment as has been done previously with other promoters that can be quickly activated. This is not possible for Zap1 target promoters because it takes several hours to induce these promoters as a cell transitions from a zinc-replete to a zinc-limited state. On other promoters where these experiments have been done, it was found that assembly is a highly ordered process (Biddick and Young, 2009). On the HO promoter, for example, Mediator is recruited by the Swi5 activator following SWI/SNF and SAGA entry (Cosma, 2002). In contrast, Mediator is recruited by Gal4 to the *GAL1* promoter after SAGA but before SWI/SNF (Bryant and Ptashne, 2003; Lemieux and Gaudreau, 2004). Zap1-mediated induction differs from that of Gal4 where SWI/SNF and SAGA are recruited by the activator independently of each other. Thus, our findings reflect the heterogeneity of coactivator interactions as observed in other coactivator recruitment studies.

If AD1 and AD2 require the same coactivators, as our data suggest, why are some promoters responsive to AD2 and other promoters are not responsive? As described above, our knowledge of what coactivators are required for function of AD1 and AD2 is still incomplete and there may be some key differences in the specific complexes recruited by these two domains. Continued analysis of coactivator recruitment by AD1 and AD2 may identify AD-specific coactivators. Our results do support a second hypothesis that is AD2 is a weaker-activation domain and may therefore be incapable of activating those genes that require a strong activation domain due to, for example, especially repressive nucleosome positioning. Promoters that are better poised for activation would then be responsive to either a strong activation domain (AD1) or a weaker domain like AD2. We showed previously that while AD1 is sufficient to activate transcription of the *ZRT1* gene under normal conditions, it was not sufficient to activate transcription at 37°C where AD2 was also required (Frey and Eide, 2011). We suggested that AD2 may therefore be needed to aid AD1 when zinc deficiency is combined with other stresses such as heat stress. Now that we know some of the coactivators recruited by Zap1, we can further explore the novel roles of AD1 and AD2 in Zap1-mediated transcription.

## Experimental Procedures

### Growth conditions

Yeast strains were grown in either YP medium supplemented with 2% glucose (YPD) or synthetic defined medium with 2% glucose and the appropriate auxotrophic supplements. Limiting zinc medium (LZM) was prepared as previously described (Gitan et al., 1998) with 2% glucose as the carbon source and the indicated concentration of ZnCl_2_. LZM contains 1-mM EDTA and 20-mM citrate as metal buffers to limit zinc availability. Because of those metal buffers, the zinc available to cells in LZM is far lower than the total concentration.

### Yeast strains and plasmids

Yeast strains used in this study included DY1457 (MATα *ade6 can1 his3 leu2 trp1 ura3*), ABY9 (DY1457 *zap1*Δ::*KanMX4*) (Zhao and Eide, 1997), BY4741 (*MATa his3Δ leu2Δ met15Δ ura3Δ*) and BY4743 **(***MATa/α met15Δ/+ lys2Δ/+ his3Δ/ his3Δ leu2Δ/ leu2Δ ura3Δ/ ura3Δ***)**. All strains newly constructed for this study are listed in Table 2. The homozygous coactivator mutant strains (Open Biosystems) were all isogenic with BY4743 and contain the corresponding gene deleted and replaced with the KanMX4 cassette. The TAP-tagged coactivator strains (Open Biosystems) were isogenic with BY4741 and had the tandem affinity purification (TAP) tag inserted at the C-terminus of the indicated gene. To delete the *ZAP1* gene in these strains, KanMX4 or HphMX4 cassettes flanked by 500 base pairs of the promoter and terminator regions of *ZAP1* were generated by overlap PCR and the resulting fragment was transformed into recipient strains to generate isogenic *zap1*Δ mutants. The *ZRT1-lacZ* and *DPP1-lacZ* reporters were previously described (Lyons et al., 2000). The *YOR387C-lacZ* reporter was constructed by amplifying the 1000 base pair region upstream of the translational start site of the *YOR387C* gene using primers with 40 bp of homology to YEp353 (Myers et al., 1986). The resulting fragment was then inserted into BamHI-, *E*coRI-digested YEp353 using homologous recombination. Plasmids pYef2L (vector), pYef2L-Zap1-6x-myc (Zap1^WT^), pYef2L-Zap1^Δ6-551^-6x-myc (Zap1^AD2^), and pYef2-Zap1^ΔZnf1/2::GliZnf1/2^-6x-myc (Zap1^AD1^) were previously described (Bird et al., 2000; Frey and Eide, 2011). These plasmids express *ZAP1* from the *GAL1* promoter. To normalize protein expression of the different myc-tagged Zap1 alleles to that of endogenous Zap1, cells were cotransformed with plasmid pGEV (Gao and Pinkham, 2000). GEV is a hybrid-activator protein containing the Gal4 DNA-binding domain, the hormone-responsive domain of the estrogen receptor, and VP16 activation domain. Treatment of GEV-containing cells with 10-nM β-estradiol resulted in expression of these Zap1 alleles at levels equal to chromosomally expressed Zap1 (Frey and Eide, 2011).

**Table 2:** Strains generated in this study

Strain	Relevant genotype^1^
AFY101	*zap1Δ::hphMX4 ada2Δ::kanMX4*
AFY102	*zap1Δ::hphMX4 ada3Δ::kanMX4*
AFY103	*zap1Δ::hphMX4 spt3Δ::kanMX4*
AFY104	*zap1Δ::hphMX4 swi3Δ::kanMX4*
AFY105	*zap1Δ::hphMX4 snf6Δ::kanMX4*
AFY106	*zap1Δ::hphMX4 rsc2Δ::kanMX4*
AFY107	*zap1Δ::hphMX4 dhh1Δ::kanMX4*
AFY108	*zap1Δ::hphMX4 paf1Δ::kanMX4*
AFY109	*zap1Δ::hphMX4 ccr4Δ::kanMX4*
AFY110	*zap1Δ::hphMX4 not5Δ::kanMX4*
AFY111	*swi3Δ::kanMX4 ADA2-TAP::HIS3*
AFY112	*ada2Δ::kanMX4 SPT3-TAP::HIS3*
AFY113	*swi3Δ::KanMX4 SPT3-TAP::HIS3*
AFY114	*ada2Δ::kanMX4 SWI3-TAP::HIS3*
AFY115	*ada2Δ::kanMX4 MED15-TAP::HIS3*
AFY116	*swi3Δ::kanMX4 MED15-TAP::HIS3*
AFY118	*zap1Δ::kanMX4 MED15-TAP::HIS3*
AFY119	*zap1Δ::kanMX4 SPT3-TAP::HIS3*
AFY120	*zap1Δ::kanMX4 SWI3-TAP::HIS3*

1 All of these strains were derived from BY4741.

### S1 nuclease protection assays

RNA was extracted from cells using hot acid phenol extraction and S1 analysis was performed as previously described (Dohrmann et al., 1992). Thirty micrograms of total RNA was hybridized to a ^32^P end-labeled oligonucleotide probe before digestion by S1 nuclease and separation on an 8% polyacrylamide/8 M urea gel. Probes are listed in Table 3.

**Table 3:** Oligonucleotides used in this study

Gene	Purpose	Sequence
*YOR387C*	S1 nuclease assay	5′-TTATTACAAGTGACGTTAGTCAAATCAAA TCTGACGGCTGCCATGGT-3′
*ZRT1*	SQ-PCR/ChIP^1^	5′-CAATACACCCGTACTCTCTTGCCTGT-3′
*ZRT1*	SQ-PCR/ChIP	5′-TGCTCTCAACCTACTTTCCATGAC-3′
*CMD1*	SQ-PCR/ChIP	5′-CCTCCAATCTTACCGAAGA-3′
*CMD1*	SQ-PCR/ChIP	5′-GCGGGAGCAAAAAATCACA-3′
*ZPS1*	SQ-PCR/ChIP	5′-GCCGTTTCTTTTTGGGCAGTA-3′
*ZPS1*	SQ-PCR/ChIP	5′-GCCTTTAAAAACAGCGCTTCC-3′
*ZRT1*	RT-PCR/ChIP^2^	5′-CGCGCGCCAGATAACTAAAA-3′
*ZRT1*	RT-PCR/ChIP	5′-ACCGCACAGATGAGAACCTTG-3′

1 Used for semi-quantitative PCR analysis of chromatin immunoprecipitation (Fig. 4).

2 Used for quantitative real-time PCR analysis of chromatin immunoprecipitation (Figs. 5 and 6).

### β-galactosidase assays

Cells were grown for 15–20 h to mid-log phase (A_600_ = 0.3–0.7) in LZM supplemented with the indicated amount of ZnCl_2_. β-galactosidase activity was measured as described (Guarente, 1983) and activity units were calculated as follows: (ΔA_420_ × 1000)/(min × mL of culture × absorbance of the culture at 595 nm).

### Immunoblot analysis

Protein extracts for immunoblots were prepared by cell disruption in the presence of trichloroacetic acid (Peter et al., 1993). Immunoblots were performed essentially as described (Harlow and Lane, 1988). Proteins were separated by SDS-PAGE (7.5% acrylamide) and then transferred to nitrocellulose. Blots were probed with anti-Zap1 (Evans-Galea et al., 2003), anti-c-myc (monoclonal 9E10, Roche), or anti-Pgk1 (Molecular Probes) antibodies, washed, and then incubated with either goat anti-mouse or goat anti-rabbit IgG antibodies coupled to horseradish peroxidase. TAP-tagged proteins were detected using goat anti-rabbit IgG antibodies. Detection was by enhanced chemiluminescence (ECL; Amersham).

### Chromatin immunoprecipitation

Chromatin immunoprecipitation was performed as described (Kanin et al., 2007). Wild-type BY4741 cells or isogenic strains with TAP tag insertions (Open Biosystems) in *ADA2*, *MED15*, *SPT3*, or *SWI3* were grown to an A_600_ ∼ 0.5 and then treated with 1% formaldehyde to cross-link protein-DNA complexes. The cross-linking reaction was quenched by adding 125-mM glycine. After two washes with ice-cold PBS, the cells were lysed with glass beads in buffer containing Complete Protease Inhibitor Cocktail (Roche), 1-mM PMSF, and 2 mM-benzamidine. Following centrifugation for 10 min at 16,000 × *g*, the supernatants were incubated with IgG-Sepharose (GE Healthcare) at 4°C for 1 h. The cross-links were reversed in TES and coprecipitation of specific promoter fragments with the TAP-tagged coactivator was assessed by PCR using primers flanking the *ZRT1* ZREs (Table 3). Primers specific to the *CMD1* promoter were used as a negative control because this housekeeping gene relies on TFIID for activation and not the coactivators assayed in these experiments. Quantitative analysis of chromatin immunoprecipitation fractions was performed using real-time PCR. The relative amount of coprecipitated DNA was calculated from input DNA and is reported as a fold-increase relative to *zap1Δ* cells containing an empty vector.
